# A Green Analytical Method Combined with Chemometrics for Traceability of Tomato Sauce Based on Colloidal and Volatile Fingerprinting

**DOI:** 10.3390/molecules27175507

**Published:** 2022-08-27

**Authors:** Alessandro Zappi, Valentina Marassi, Nicholas Kassouf, Stefano Giordani, Gaia Pasqualucci, Davide Garbini, Barbara Roda, Andrea Zattoni, Pierluigi Reschiglian, Dora Melucci

**Affiliations:** 1 Department of Chemistry “Giacomo Ciamician”, University of Bologna, 40126 Bologna, Italy; 2 byFlow srl, 40129 Bologna, Italy; 3 COOP ITALIA Soc. Cooperativa, Casalecchio di Reno, 40033 Bologna, Italy; 4 CIRI Agrifood, University of Bologna, 47521 Cesena, Italy

**Keywords:** green analytical methods, asymmetric flow field-flow fractionation AF4, AF4-multidetection, food colloids, volatile compounds (VOC), Gas Chromatography, ion-mobility spectroscopy, chemometric analysis, principal component analysis (PCA), FFF-chemometrics

## Abstract

Tomato sauce is a world famous food product. Despite standards regulating the production of tomato derivatives, the market suffers frpm fraud such as product adulteration, origin mislabelling and counterfeiting. Methods suitable to discriminate the geographical origin of food samples and identify counterfeits are required. Chemometric approaches offer valuable information: data on tomato sauce is usually obtained through chromatography (HPLC and GC) coupled to mass spectrometry, which requires chemical pretreatment and the use of organic solvents. In this paper, a faster, cheaper, and greener analytical procedure has been developed for the analysis of volatile organic compounds (VOCs) and the colloidal fraction via multivariate statistical analysis. Tomato sauce VOCs were analysed by GC coupled to flame ionisation (GC-FID) and to ion mobility spectrometry (GC-IMS). Instead of using HPLC, the colloidal fraction was analysed by asymmetric flow field-fractionation (AF4), which was applied to this kind of sample for the first time. The GC and AF4 data showed promising perspectives in food-quality control: the AF4 method yielded comparable or better results than GC-IMS and offered complementary information. The ability to work in saline conditions with easy pretreatment and no chemical waste is a significant advantage compared to environmentally heavy techniques. The method presented here should therefore be taken into consideration when designing chemometric approaches which encompass a large number of samples.

## 1. Introduction

In the previous few decades, European consumers have become more aware of the importance of the quality and origin of food products. A study by the European Consumer Organisation in 2014 [1] revealed that 70% of European citizens and 82% of Italian consumers care about the geographical origin of food and that they rely on this information when making consumer choices. Consequently, food industries and consumer organisations have started paying more attention to quality standards and the geographical traceability of food products. In particular, Italy is one of the major producers and exporters of several agri-food products and remains at the forefront of consolidating, controlling, and protecting national agri-food industry [2,3,4].

There are several European and Italian regulations that aim at ensuring quality standards and label conformity. Food labelling regulations are becoming increasingly stringent in terms of permitted ingredients (type and quantity) [5,6], geographical origin, and clarity of information.

Tomato products are used all over the world and it is estimated that 30% of global production of tomato fruit is destined for industrial processing. The main producers are the United States, Italy, and China, covering together 56% of world tomato production [7]. Italian tomato sauce is protected by Ministerial Decree number 57, 2006 (DM 57/2006) [8] that states that tomato sauce must be prepared with strictly fresh tomatoes in order to avoid the addition of tomato concentrate imported from foreign markets. In addition to the quality requirements, DM 233/2017 (updated by DM 170/2020) [9] imposes the indication of geographical origin on labels. It is mandatory to indicate both the country where the tomatoes were grown and the country where the product is processed.

However, despite this extensive regulation of the production of tomato derivatives, cases of product adulteration still affect the market. Some well-known adulterations are the addition of food colourants [10], flavourings (such as paprika), use of tomato coming from countries that are not indicated on the product’s label, and use of non-compliant procedures [11]. The detection of such counterfeits is usually controlled by document control as required, for example, in the PDO (Protected Designation of Origin) regulations. However, analytical research in the agri-food field is developing methods which compare authentic and suspect samples, using several analytical [12,13,14] and statistical methods [15,16].

To control the authenticity of tomato sauce and evaluate and quantify the presence of additives and adulterations, the most used analytical techniques are high pressure liquid chromatography (HPLC) [10] and gas chromatography (GC) [17], generally coupled to mass spectrometry (MS) [17,18]. For the analysis of the tomato origin, heavy metal analysis with thermal ionisation MS (TIMS) [19] or high resolution inductively coupled plasma MS (HR-ICP-MS) [17,20] has been utilized. However, most of the cited techniques require several chemical pretreatments on the samples before analysis [10,17], making each analysis expensive (e.g., solvents are often needed to extract the fraction which will be analysed) and time-consuming. Moreover, HPLC often requires the use of organic solvents. In this paper, a green, low-cost analytical procedure has been developed for the analysis of tomato sauce via statistical analysis of volatile organic compounds (VOCs) [21] and colloidal fractions. The aim of this work is to analyse both of these aspects of tomato sauce using techniques that do not require sample pretreatment, thereby reducing analysis time and cost. For VOC analysis, the work was carried out using head-space (HS) GC coupled both to a flame ionisation detector (GC-FID) [22] and to an ion mobility spectrometer (GC-IMS) [23,24].

The profile of VOCs within tomato sauce contains a great number of different molecules, although not all equally contribute to the taste and aroma [25,26,27]. Aldehydes and alcohols constitute the most concentrated classes [26] but there are also ketones, hydrocarbons, esters, and nitrogen- and sulphur-containing molecules. The presence and concentration of such VOCs depend on the tomato species, the portion of the fruit (pericarp, septa, columella, locular gel and seeds, and stem end [26]), and ripening degree. Their origin is due to the action of endogenous enzymes (in particular lipoxygenase and glycosidase) that oxidate larger molecules (e.g., terpene and carotenoids) to produce VOCs [28]. Colloidal particles instead originate from the presence and interactions of macromolecules as proteins (averaging 17% *w*/*w*), polysaccharides, condensate polyphenols, and other less abundant compounds [29]. They influence the physio-chemical properties of tomato sauce including shelf life, and taste sensory perception. Their content can greatly vary depending on ripening stage and processing [30,31]. Both the volatile profile and composition of the colloidal fraction depend on tomato cultivar and growing conditions [32], ripening stage [33], transport [28], storage, and processing methods. The combination of these factors can result in specific fingerprints based on volatile and colloidal profiles which provide a useful resource for the characterization of the product via rapid, non-destructive sampling methods. In fact, current industry practice for detecting product fraud involves the creation of a screening-model based on fingerprints of genuine products against which the fingerprint of a suspect sample is compared. This methodology allows the producer to assess whether or not the sample belongs to the same class as the training set.

The colloidal fraction of tomato paste was analysed by asymmetric flow field-fractionation (AF4). This AF4 is a soft separation technique able to size-sort colloidal dispersions according to the hydrodynamic size of particles. Separation is performed in the absence of a stationary phase which avoids particle alteration [34,35,36]. Carrier fluids, pH, and salinity can be adjusted to match the required environment, while the separation device geometry allows for simultaneous filtration of ions and small molecules thus leading to the selective characterization of the colloidal particles [34,35,37,38]. Moreover, AF4 multi-detection platforms can include various detectors such as UV-Vis, fluorescence, and multi-angle light scattering (MALS) to provide sample composition and spectroscopic properties, monitor stability, and investigate aggregation and conjugate formation [39,40,41]. To date, AF4 has been employed on numerous samples such as biocompatible nanoparticles [42,43], plant-derived proteins, and biological samples in the native state [44,45,46,47]. In the field of food and ingredient analysis, AF4 has been used to characterise the macromolecular and colloidal fractions of wine [48,49,50,51], milk [52,53], and caseins [54]. Some chemometric approaches using AF4 data were reported [55,56]. However, so far AF4 has not been applied to tomato sauce assaying, except for studies concerning plastic contamination in food matrices [57], and no attempts at chemometric classification with FFF data are reported yet: to the authors’ knowledge, it is the first time that the colloidal fraction of tomato sauce has been studied and characterised by AF4 multi-detection and that FFF data from tomato sauce has been subjected to chemometric analysis.

An important trait of this approach is that the only chemicals required for the entire experimental setup described are limited to the AF4 mobile phase, which can be a saline solution, making the analysis rapid, non-toxic, and solventless.

Datasets from GC and AF4 have been analysed by chemometric methods in order to understand the fundamental characteristics of tomato sauce. Different data elaborations of GC and AF4 results showed promising grouping perspectives, confirming that it could be a valuable approach in wider projects aimed at finding methods able to discriminate the geographical origin of food samples, and to identify counterfeits or adulterations [58,59,60]. With different elaboration approaches, AF4 data yielded comparable or better results than GC-IMS in terms of quality, and also offered complementary information. Moreover, the use of untargeted methods based on chemometrics makes it possible to focus attention on a limited number of variables for future discrimination [61], without the need for a full screening test for all of the compounds present in tomato sauce.

## 2. Materials and Methods

### 2.1. Tomato Sauce Samples

Forty-six tomato sauce samples were purchased for this study. All samples were labelled as “100% Italian” products, and the commercial name was hence “100% Italian tomato sauce”. Samples were purchased after their distribution in supermarket chains. The sampling campaign concerned 29 different commercial brands and 21 different manufacturers. Indeed, it is common for manufacturers (i.e., companies growing and/or processing tomato) to produce tomato sauce for more than one brand (i.e., the brand on the label) which then retails it under their own name. For six commercial brands and seven manufacturers, more than one sample was purchased (6 for brand 1, 2 for all of the other brands). These are the main commercial brands in Italy and most of the attention was focused on them. For each of the other brands, only one sample was purchased, and all samples were used as a “bulk” to which the six main brands were compared.

### 2.2. Samples Preparation and Analysis

All samples were kept closed in their original package (glass bottle) until analysis. At the point at which the package was opened, sample preparation was carried out for all the three analytical methods used in this work (GC-FID, GC-IMS, and AF4 multi-detection). The common trait of all the analytical methods is that no chemical pretreatment is required on the samples, with the advantages of reducing preparation time and cost.

#### 2.2.1. GC-FID Method

An unaltered aliquot of 2 g (±1%) of sample product was placed in a 20-mL vial, which was immediately sealed with an air-tight cap and then placed in the auto-sampler of the instrument.

Samples were then analysed with a Heracles II gas-chromatograph (Alpha MOS, Toulouse, France). Temperature was set at 50 °C for 20 min, shaking at 500 rpm, to concentrate volatile compounds in the headspace of the vial. Then, a 5 mL aliquot of the headspace was sampled with a syringe and adsorbed on a CARBOWAX trap (40 °C for 65 s) located before the chromatographic columns. Analytes were then desorbed by increasing the temperature up to 240 °C, and transported by the carrier gas (H_2_) into the chromatographic column. Heracles II contains two columns working in parallel: a non-polar column MXT-5 (5% diphenyl-polysiloxane and 95% methyl-polysiloxane) and a slightly polar column MXT-1701 (14% cyanopropilphenyl-86% methyl-polysiloxane). Both are 10-m long and have an internal diameter of 180 μm. A valve splits the sample into equal parts and controls entry into the two columns of the volatile compounds after desorption. The temperature was initiated at 40 °C and increased to 270 °C at 3 °C s^−1^. The total time for a single analysis was 100 s, with data collected at an interval of 0.01 s by a FID detector.

The two chromatograms obtained by the two columns were appended to each other into a single chromatogram. Chromatograms were processed by AlphaSoft v.12.44 (Alpha MOS, Glen Burnie, MD, USA), which automatically integrates the chromatogram peaks and transcribes peak-areas in a data matrix. Samples were replicated twice. No identification or quantification of the volatile molecules was carried out, but the peak areas were used in an untargeted way to perform chemometric analyses.

#### 2.2.2. GC-IMS Method

The sampling procedure is analogous to that described for GC-FID. An unaltered aliquot of 2 g ± 1% of sample product was placed in a 20-mL vial, sealed with an air-tight cap, and placed into the auto-sampler of the instrument. The analysis was carried out with a FlavourSpec (GAS Dortmund, Dortmund, Germany) gas-chromatograph. This instrument holds a FS-SE-54-CB column (94% methyl-5% phenyl-1% vinyl-polysiloxane), 60-m long, with an internal diameter of 250 µm. The vial temperature was kept at 40 °C for 8 min. Then, 0.5 mL of headspace was sampled with a syringe and injected into the column. The carrier gas was N_2_. The temperature of the chromatographic column was kept constant at 40 °C for the entire analysis time (34 min). The carrier gas flow was kept at 2 mL min^−1^ for 2 min, then increased to 17 mL min^−1^ over 6 min and kept constant for 12 min, then decreased to 2 mL min^−1^ over 12 min and kept for 2 min.

The detector of this instrument is a drift tube, 98 mm long, in which the analytes outgoing from the chromatographic column are ionised at 5000 V and subjected to an electric field. Ionised analytes are pushed toward a Faraday plate that detects them. A drift gas (N_2_) flows in the opposite direction. In this way, analytes are further separated within the drift tube, reaching the Faraday plate at different times (drift time). The difference in drift times is based on ion mobility, which is influenced by mass, dimension, shape, charge, and by the collision cross section between the drift-gas molecules and ions. The temperature of the drift tube was kept at 45 °C.

The result of the analysis is a 2D-graph in which the vertical axis is the result of the chromatographic run, while the horizontal axis reports the drift time of IMS. The software connected to the instrument, Laboratory Analytical Viewer (GAS Dortmund), automatically integrates the chromatogram peaks and transcribes peak-areas in a data matrix. Samples were replicated twice. No identification or quantification of the volatile molecules was carried out *a priori*, but the peak areas were used in an untargeted way to perform chemometric analyses. A tentative identification of discriminating compounds was carried out after chemometric analyses, as described in paragraph 3.2, by comparing retention times and drift times of the 2D peaks with the FlavourSpec database using the VOCal v.0.1.0 software (GAS Dortmund).

#### 2.2.3. Analysis by AF4

Tomato sauces were previously centrifuged at 13,400 rpm for 30 min to remove micro and millimetric matter. Subsequently, the supernatant was filtered by a 45 μm syringe filter.

**Figure 1:**
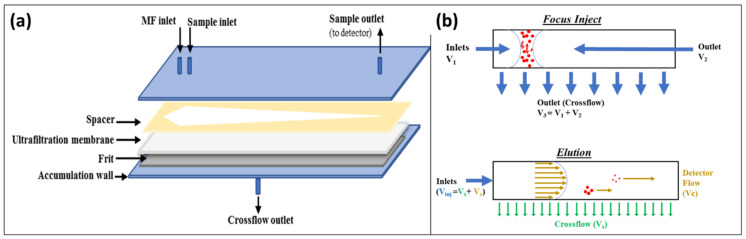
Schematics and working mechanism for AF4: (**a**) schematization of the AF4 channel used for the experiments; and (**b**) schematization of the two main steps of the *Separative Experiment*, channel represented as a longitudinal cross section.

Separation by AF4 is performed in an empty trapezoidal channel and at room temperature. The channel consists of an ultrafiltration membrane of a suitable material (such as cellulose or Poly Ether-Sulfone (PES) placed on a spacer with a typical thickness of 250–800 µm. A porous frit of ceramic or metal material is placed under the filter membrane (accumulation wall) and the assembly is confined between polycarbonate walls. A schematic of the channel is detailed in Figure 1.

An AF4 *separative experiment* is composed of two principal steps: focus (injection), and elution (Figure 1b). During the *focus-injection* step, analytes are equilibrated in a narrow band at the beginning of the channel. In the elution step, the flow (V_inj_) is split in two components, a longitudinal laminar flow (with a parabolic profile) named *detector flow* and a perpendicular flow named *crossflow*, driving separation. Nano systems exhibiting colloidal behaviour are separated based on their diffusivity (inversely correlated to their hydrodynamic radius, *r_h_*) and on their interaction with the crossflow. Analytes with lower diffusivity (thus higher *r_h_*) tend to accumulate near the accumulation wall, while smaller nano systems diffuse towards higher laminar flows. In addition to the separative AF4 analysis, we conducted two other non-separative experiments: Flow Injection Analysis (FIA), and Focus-FIA (FFIA) [41,42]. An FIA is a shorter, non-separative, non-filtering analysis where the signal is related to the whole sample content. A Focus-FIA is an FIA with a preliminary focusing step. In a Focus-FIA, components smaller than the membrane cut-off are filtered out, and only the remaining colloidal part of the sample reaches the detector. The ratio between the areas of the FIA peak and the FFIA one (% FFIA/FIA) accounts for the total colloidal content of the sample.

The separation of the colloidal fraction was carried out with an AF4 Agilent 1100 system (Agilent Technologies, Palo Alto, CA, USA) combined with an Eclipse 3 Separation System (Wyatt Technology Europe, Dernbach, Germany). The channel was 152 mm long, 16 mm wide, and 350 μm thick. The membrane was made by PES with 5 kDa cut-off (Microdyn-Nadir, Wiesbaden, Germany). The mobile phase was a NaNO_3_ 62 mM solution in ultrapure water, simulating salinity of tomato sauce in order to avoid colloid modifications during separation and analysis. The coupled detectors were a diode-array UV/Vis spectrophotometer, a fluorimeter, and a MALS detector.

The detector flow rate was 0.60 mL min^−1^ for FIA, Focus-FIA and the separation method. The injection flow rate was 0.20 mL min^−1^ and the *Focus-injection* step was 8 min long with a 2.0 mL min^−1^ crossflow rate both for Focus FIA and method. During the elution step of the separation method, the crossflow decayed exponentially from 2.5 mL min^−1^ to 0.12 mL min^−1^ over 25 min, and remained constant for 17 min before field release.

The precision of all the methods was assessed both on retention times and on signal intensity by performing three independent replications (both intra- and inter-day) for each tomato sample used to develop the methods. The profiles exhibited a maximum of 0.5% and 1% deviation in terms of retention time and signal intensity, respectively. Sample injection volume was 300 μL for FIA, FFIA and AF4 analyses. Samples were analysed in triplicate.

### 2.3. Principal Component Analysis

Principal component analysis (PCA) [62] was performed on all GC and AF4 data. The GC data are the peak areas as calculated by the software of both Heracles II and FlavourSpec GC. The AF4 data used here consisted in both the full profiles obtained by the UV-Vis detector (Supplementary Materials, Figure S1a) and the peak areas calculated from said profiles as well as those obtained from FIA and FFIA analyses as described in Section 2.2.3. All data were used in an untargeted way. The PCA is a well-known chemometric procedure that provides dimensionality reduction and visualisation of data. It rotates the original variables into a new reference space oriented along the dimensions that best describe the variance within the data. The versors of this new space are the principal components (PCs), the scores are the coordinates of objects (samples) in PC space, and the loadings are the coordinates of variables in PC space. For the present work, the PCs considered relevant and shown in the results were those reporting at least 10% of explained variance. Chemometric analyses were performed by the software R v.4.1.0 (R Core Team, Vienna, Austria).

## 3. Results and Discussion

### 3.1. The GC-FID Method

**Figure 2:**
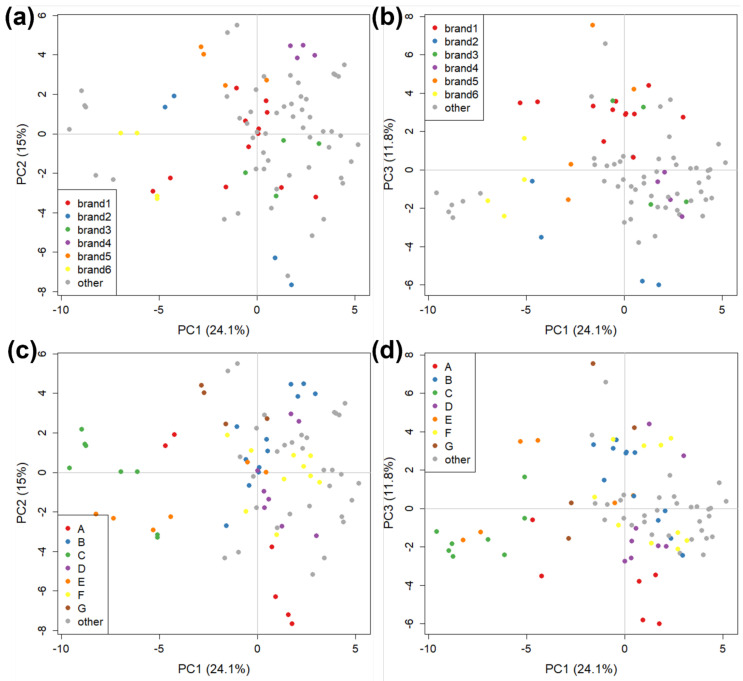
The PCA score plots on GC-FID data (without outliers) divided by: (**a**,**b**) brand name (i.e., the brand name of the retail product); and (**c**,**d**) manufacturers (i.e., the plant where the tomato sauce is manufactured). (**a**,**c**): PC1 vs. PC2; (**b**,**d**) PC1 vs. PC3.

The GC-FID data were used as a screening test with all samples to check for possible differences between the most represented brands (from 1 to 6) and manufacturers (A to G), and to compare them with the other brands, for which only one sample was purchased. The starting dataset consisted of 92 objects (two replicates of each tomato sauce sample) and 61 variables, corresponding to Heracles II integrated peak areas. For all PCAs on GC-FID, data were auto-scaled (i.e., the column mean is subtracted to each point and the result divided by the column standard deviation) before the analysis. A first explorative PCA showed the presence of three samples (six objects) that were very different from the others. These samples fell outside of the Hotelling ellipsoid [63], corresponding to 95% confidence level, and hence were considered outliers and removed from the dataset before further analysis. The PCA was repeated without the outliers and Figure 2 shows the score plots obtained. The relative variance carried by each PC is 24.1% for PC1, 15% for PC2, and 11.8% for PC3 (50.9% of total explained variance). Despite carrying more than 10% explained variance, PC3 is similar to PC2 and so is also considered when evaluating the model.

Figure 2 shows that all samples, excluding the outliers, form a homogeneous group around the centre of the score plot. Little significant difference is observed between the VOC fraction of commercial tomato sauce samples, although it is possible to observe some degree of clustering both by brand and by manufacturer. Each manufacturer can produce tomato sauce for more than one brand (which then retails it under their own name and label), but it can also employ different production lines according to the brand. Therefore, it is important to consider both aspects for each sample when attempting to discriminate between products. Brand 1 (the most represented brand, in red) and 4 (in violet) are well grouped both in PC1 vs. PC2 and PC1 vs. PC3 (Figure 2a,b). The other major brands show less or no clustering at all (e.g., brand 3, in green) in at least one of the two plots. Separation by manufacturer is more evident with clusters better defined in both plots (Figure 2c,d), except for one sample each from manufacturer A (in red) and E (in orange) that are far from the others. Samples clearly show more similarities when they are produced by the same manufacturer compared to samples that are sold by the same brand, which is reasonable since the main difference in product should be due to local origin and processing rather than labelling. In particular, manufacturer C shows a distinct cluster which is placed at negative values of PC1, far from other samples. Heracles II does not provide identification of the chromatographic peaks [56]. Therefore, at this stage of the study, no more chemical information from the loading plots about the differences between brands and manufacturers could be provided.

**Figure 3:**
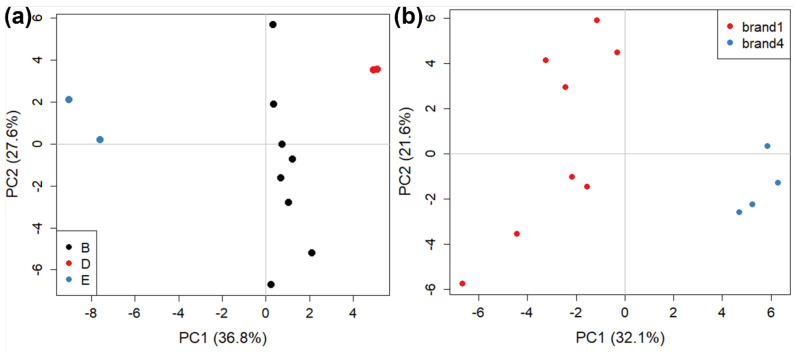
The PCA score plots (PC1 vs PC2) describing: (**a**) anufacturer (i.e., the plant where the tomato sauce is manufactured) discriminations for brand 1, and (**b**) brand (i.e., the brand name of the retail product) discrimination for manufacturer B.

We then examined the possibility of further discriminating between samples sold by the same brand but produced by different manufacturers, and between samples produced by the same manufacturer but sold by different brands, which can help identify if a specific brand requires specific processing steps, for example. We focused our attention on two different subsets of our data. The first subset was composed of six samples, all sold by brand 1 and produced by three different manufacturers (B, D, E). The score plot for PC1 vs PC2 contained 52.4% explained variance, shown in Figure 3a. The clustering based on different manufacturers is readily apparent along PC1, where they are well discriminated, although only one sample (but two replicates) were present for manufacturers D and E. This is further evidence that the differences due to manufacturing are stronger than those due to commercial brands. The second subset considered contained six samples, produced by manufacturer B and sold by two different brands (1 and 4). The score plot for PC1 vs PC2 contained 47.7% explained variance and is shown in Figure 3b. In this case, the discrimination is good, indicating that the manufacturer is likely to use different production lines for the different brands.

**Figure 4:**
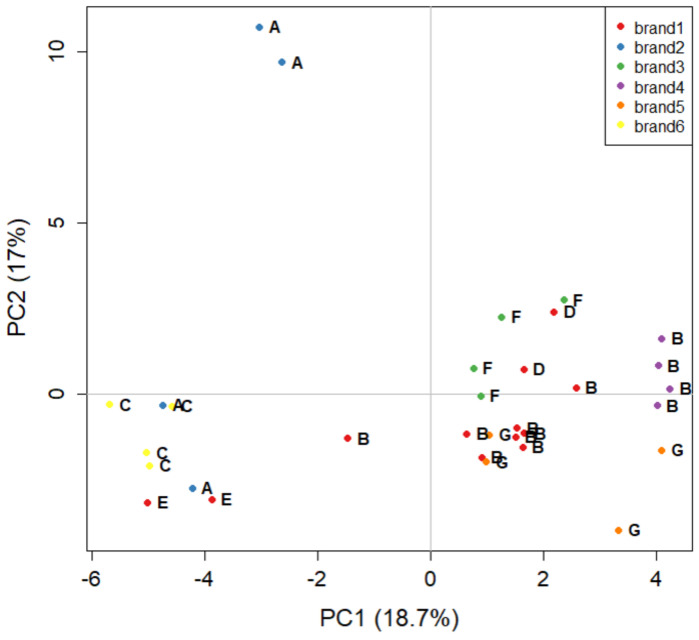
The PCA score plot of the five major brand names of tomato sauce labelled by colour. Letters indicate the corresponding manufacturer (i.e., the plant where the tomato sauce is manufactured).

The last PCA carried out on the GC-FID dataset is focused only on the six major brands (16 samples, 32 objects): Figure 4 shows the corresponding score plot, carrying 35.7% of the explained variance. Figure 4 presents further evidence that the manufacturer is more easily discriminated than the brand. Samples from manufacturer B are all concentrated at positive values of PC1 (except for a replicate of brand 1), regardless of belonging to brand 1 or 4. The only exception is brand 2, the samples of which are both produced by manufacturer A, but are very well separated in the score plot. The distance of the sample at high positive values of PC2 could suggest that it is an analytical outlier, possibly produced in a batch with strong differences relative to all of the other ones. In fact, both replicates of this sample are close to each other, strengthening the hypothesis that this sample is an outlier.

Overall, the chemometric analysis of head-space GC-FID analysis was used to identify outliers and homogeneous clusters, discriminate between different manufacturers for the same brand, and identify well-defined clusters of products from the same manufacturers produced for different brands.

The characteristics of this analytical method, such as easy and cheap sample preparation, high speed of analysis (100 s), and rapid visualisation of results makes it a valid screening test and a possible alternative to traditional analyses of tomato sauces. Moreover, the simultaneous use of two chromatographic columns drastically increases the quantity of information that can be obtained from a single sample. However, at this stage it is still not possible to reliably identify the VOCs analysed by Heracles II due to lack of databases for tomato sauce. Future development should aim to expand the technique to facilitate the identification of specific VOCs that are responsible for the differences between samples.

### 3.2. The GC-IMS Method

**Figure 5:**
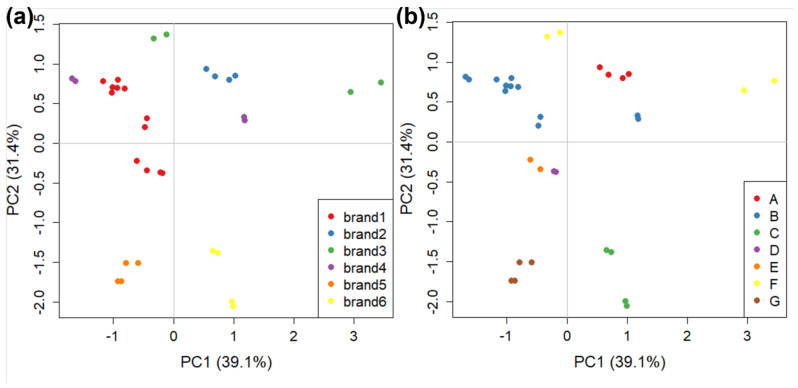
The PCA score plots on GC-IMS data obtained for tomato sauce divided by: (**a**) brand names (i.e., the retailer name on the label); and (**b**) manufacturers (i.e., the plant where the tomato sauce is manufactured).

The CG-IMS analysis is much slower than the GC-FID one (34 min compared to 100 s). Therefore, we decided to focus on only the six major brands (1 to 6, 16 samples in total). The 2D output of GC-IMS analysis (Section 2.2.2) is automatically converted into a vector by the software instrument. This is achieved by imposing a 20 × 19 grid on the graph and then, for each square, calculating its maximum and using this as the corresponding vector value. In this way, each object is represented by a 380-length vector. Work is still in progress to optimise the use of GC-IMS with chemometrics, but this goes beyond the scope of the current work. The dataset is composed of 32 objects and 380 variables, and data were centred before PCA analysis. The PCA score plot with all samples for this analysis (70.5% of explained variance in PC1 and PC2) is reported in Figure 5, with samples divided both by brands and by manufacturers.

In general, Figure 5 shows good clustering both by brand and by manufacturer, except for the samples belonging to brand 3 and brand 4. In these cases, two samples for each brand were analysed. Despite coming from the same manufacturer, they were strongly separated in the score plot.

The visualisation of loadings is not useful due to the way in which variables are calculated by the software. Loadings indicate the values calculated for the 380 squares of the grid used by the software instead of specific peaks. However, each square can contain one or more GC-IMS peaks, therefore knowing the correlation between the square of the grid and the position on the 2D plot coming from the GC-IMS analysis, the operator can return to specific peaks included in specific squares indicated as discriminated by the loading plot [64]. In order to explore the factors that led to samples of brands 3 and 4 being distant in the score plot of Figure 4, we calculated a “partial” PCA with only the interested samples (data not shown). The loadings indicated the IMS squares which contributed the most to the discrimination between the analysed samples. Tentative attribution to specific molecules can therefore be made for the peaks present in these areas. This was performed by comparing the combination of chromatographic retention time and drift time with the library included in the IMS software VOCal v.0.1.0. This analysis was also performed on one sample of brand 1 and one sample of brand 6 (at the opposite sides of the score plot in Figure 5).

**Figure 6:**
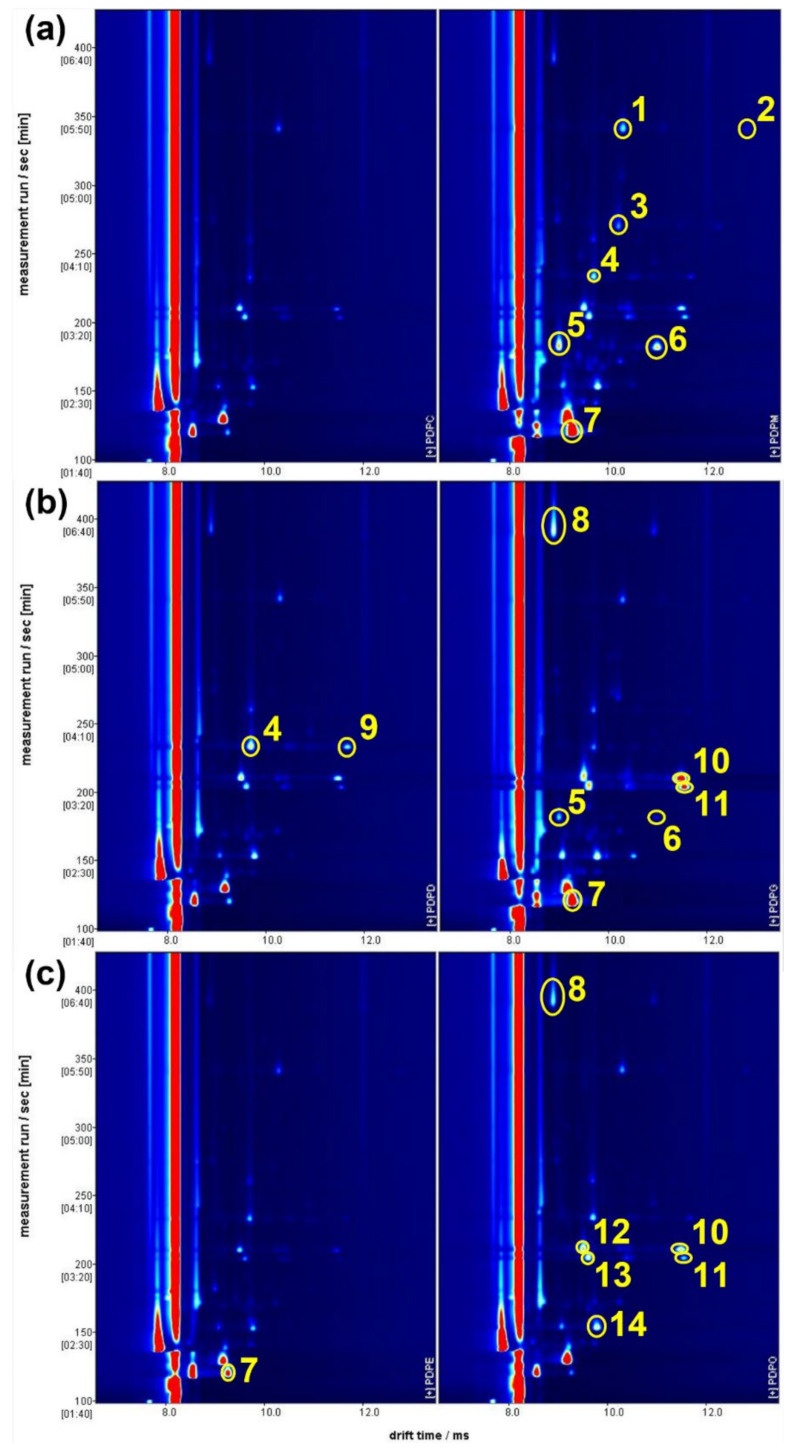
**The** GC-IMS 2D plot comparing the volatile content of three pairs of tomato sauce samples: (**a**) one sample from brand 1 and one sample from brand 6; (**b**) two samples from brand 3; and (**c**) two samples from brand 4. Abscissas report the drift times, while ordinates report the retention times. Samples in the left side are at negative values of PC1 in Figure 4. Yellow circles indicate the most discriminative peaks. The peak numbers are referenced to their corresponding molecule attribution in Table 1. Molecules are highlighted only in the sample in which the peak is most evident.

The GC-IMS 2D plots generated were then extracted and analysed, guided by the loadings obtained from the previously described PCA. These 2D plots are shown in Figure 6, with the characteristic peaks highlighted. These peaks pertained to compounds which are present in both samples but with different concentrations and peak intensities. For each pair of samples, the characteristic peaks have only been highlighted in the sample in which they are most concentrated. The distribution of these peaks suggests that the samples with positive values of PC1 in Figure 6 (in the right hand section of Figure 6) are those in which the VOCs are more concentrated. A tentative compound attribution for the highlighted peaks was performed, and reported in Table 1. Most of the identified molecules were already found in the volatile fraction of fresh tomato or tomato sauce, both as natural compounds contributing to tomato aroma and as secondary products of tomato processing. To the authors’ knowledge, only 1-hexene has not previously been reported in tomato sauce; however, it is not clear if it is a false attribution, or if its presence is due to tomato processing.

### 3.3. The AF4 Method

**Figure 7:**
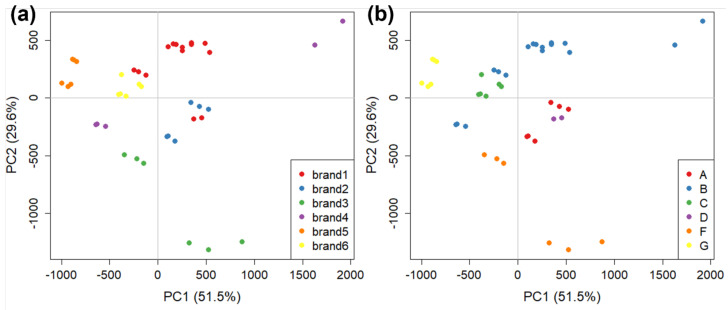
PCA score plots on AF4 data obtained for tomato sauce colloidal fraction divided by: (**a**) commercial brands (i.e., the retailer name on the label); and (**b**) manufacturers (i.e., the plant where tomato sauce is manufactured).

Similarly to the aforementioned GC-IMS method, in the case of AF4, analyses were also focused on the 16 samples representing the major brands, and three replicates were carried out for each sample, excluding one sample (manufacturer E) which could not be analysed. Therefore, for AF4 we had a total of 45 objects. The AF4 profile of tomato samples were variable in shape but always contained three bands (Supplementary Materials, Figure S1a) visible both through UV-VIS absorption and fluorescence; the absorption spectrum between 190 and 700 nm was collected for each sample to explore possible diagnostic signals (Supplementary Materials, Figure S1b). Fluorescence emission, which was tuned to proteins, confirmed that protein presence reflected the three bands observed. The size distribution of eluted particles was also evaluated with MALS, confirming that we could observe small, aggregated, and highly aggregated protein systems (Supplementary Materials, Figure S2). Given that the absorption profile remained constant along the fractogram, absorption at 280 nm vs. analysis time was chosen for its sensitivity—primarily due to the lack of interference—and was consequently used as a fingerprint. This signal was cut at the beginning and at the end due to erroneous readings. Therefore, the signals used for chemometric analyses ranged from 9.0 to 50.0 min only, including the elution time cleared of system peaks. The total number of variables was 6245. Variables were centred before chemometric analyses. A further PCA was then performed, and the results reported in Figure 7. The score plot of PC1 vs. PC2 carries 81.1% of explained variance. In this case, both brands and manufacturers are well discriminated between, except for the three replicates of a sample of brand 1 (in red) that deviate from the bulk of the others. This sample is produced by a different manufacturer compared to the others, which is likely the cause of the deviation. In Figure 7a, there is a significant difference between two sets of samples representing brand 4 (in purple). This is a similar difference to the one observed with CG-IMS (paragraph 3.2, Figure 5); however, in that case, the volatile fraction had been analysed, while with AF4 the tomato sauce bulk was analysed. This is an indication that the two techniques can be used in a complementary way for food analysis, obtaining similar results despite the portion of the food matrix that is analysed being different. A similar behaviour can be observed also for brand 5 (and manufacturer F). Both in Figure 5 and Figure 7, it can be seen that there is significant distance between the two samples (it is more evident for GC-IMS), which shows a possible difference between the two despite them both belonging to the same brand and coming from the same manufacturer.

**Figure 8:**
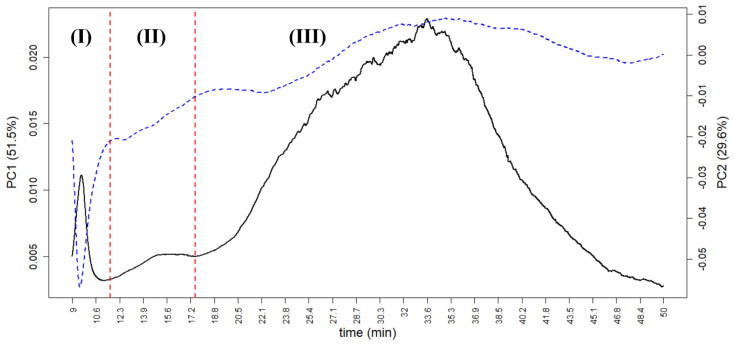
The PCA loadings on AF4 data obtained for tomato sauce colloidal fraction of PC1 (black continuous line, range on the left) and PC2 (blue dotted line, range on the right) vs. analysis time. Red dotted lines indicate the separation between three peaks, (**I**), (**II**), and (**III**), corresponding, respectively, to free proteins, small aggregates of proteins, and large colloidal aggregates.

In the case of AF4, the loading plot can give useful information about the colloidal portion of tomato sauce. The PC1 loadings, in particular, are correlated with the “mean” AF4 profile of all samples due to the high variance carried by such a PC (51.5%). Figure 8 shows the loading plot of the first two PCs based on the UV-VIS profile, in which three peaks for both PC1 and PC2 are visible, marked as (I), (II), and (III), and divided by the red vertical lines. At low time analysis (from 9.0 to 11.6 min), peak (I) represents the free proteins, the smaller portion of tomato sauce colloids; its loading is very high (by absolute value) especially in the negative part of PC2, indicating that small proteins are more concentrated in samples at negative values of PC2 than in the ones of brand 3 and manufacturer F (as seen in Figure 5). Peak (II) (from 11.6 to 17.5 min, which is less noticeable in the loadings, but sharp for some samples) represents small aggregates of proteins. Peak (III) (from 17.5 to 50 min, at the end of the *Elution* step) represents large colloidal aggregates. It shows a sharp peak at positive values of PC1, indicating that this peak discriminates the samples along PC1 in the score plot. Therefore, most of the samples from brands 1 and 2 and from manufacturers A, B, and D can be characterised by large colloidal aggregates.

**Figure 9:**
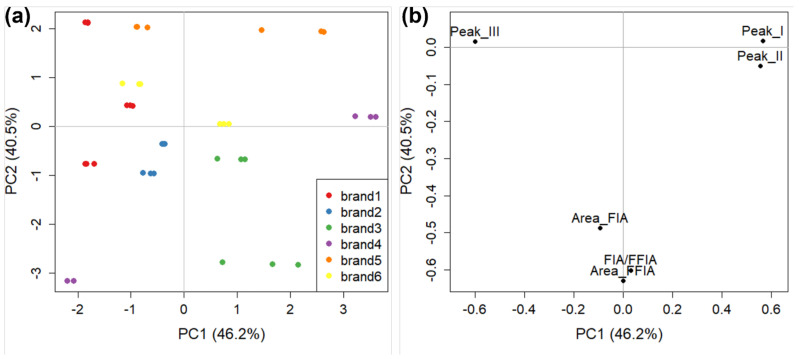
**The** PCA of FIA, FFIA, and peak area dataset obtained in AF4 for tomato sauce colloidal fraction: (**a**) score plot (with points coloured by commercial brand); and (**b**) loading plot.

To gather additional information from the AF4 separative experiment, we calculated the areas of the peak intervals for each sample and divided them by the total AF4-profile area. This produced three variables for each sample that are representative of the percentage of each colloidal fraction. These variables were joined to FIA and FFIA variables to create a new dataset. The FIA and FFIA variables are: (i) FIA peak-areas; (ii) Focus-FIA (FFIA) peak-areas; (iii) the percentage ratio between FIA and FFIA peak-areas. These three variables correspond to total content, total colloidal content, and percentage of colloidal content, respectively. This produced a new dataset that is composed of six variables that fully summarise the colloidal fraction of tomato sauces evaluated by AF4. A PCA was then performed, auto-scaling the data before analysis. In this PCA (not shown), significant distance was observed between all replicates of the two samples from brand 1 and other samples, meaning that they fell outside of the Hotelling ellipse [63] calculated for the scores. This is likely to be because FIA and FFIA analyses on these samples were carried out several days after the bottles were first opened and so it is likely that the smaller colloidal particles aggregated to form larger particles that were detected by the technique, highlighting these two samples as outliers. This demonstrates that the AF4 technique can be employed to evaluate small changes in food matrices and can potentially provide meaningful information about critical product parameters such as the shelf-life of the product. However, to better evaluate the behaviour of the other samples, those two objects were removed from the dataset and a further PCA was carried out with the others. Results (scores and loading plots) are shown in Figure 9: together, PC1 and PC2 carried 86.7% of explained variance.

Sample behaviour (Figure 9a) is very similar to that already observed for full-fractogram analysis (Figure 7), with a generally good grouping of all brands (the same for manufacturers, data not shown) except for brand 4, the two samples of which are again at the opposite sides of the PC1. The loading plot (Figure 9b) carries some information about the colloidal fraction of tomato sauce samples in a more approachable way compared to that obtained from the full fractograms (Figure 8). It is interesting to note the strong correlation between the (percentage) areas of peaks (I) and (II) at positive values of PC1, representing the smallest fraction of tomato sauce colloids, and their strong anti-correlation with peak (III) at negative values of PC1. This indicates that in most of the samples, there is a prevalence of either small or large (e.g., brand 1) colloidal species, and that the two can coexist only in a minority of samples close to the origin of the score plot. The difference between the two samples of brand 4 in this case is due to the high percentage of small particles for the sample at positive PC1 values (~80% on average, as the sum of peak (I) and peak (II)) and the high percentage of gross particles in the sample at negative PC1 values (~92%). The FIA, FFIA, and their ratio also carry similar information. The percentage of explained variance of PC2 (40.5%) is close to that of PC1 (46.2%), thus the information of FIA and FFIA is almost as relevant as that carried by the three peak areas for the sample grouping. This further validates the theoretical approach which guided the definition of these parameters, and indicates that an approach using colloidal parameters of tomato sauce can be very effective in discriminating between different brands and manufacturers.

### 3.4. Comparison with Previous Works

Due to the high commercial value of tomato sauce, several studies have already dealt with the problem of certifying its origin and its authenticity. Lo Feudo et al. [71], for example, evaluated its origin (Italian, Italian regions, and non-Italian) by ICP-MS, quantifying the concentration of 32 elements. Two more studies focused their attention on quantifying possible tomato sauce adulterations by NIR spectroscopy and electronic tongue [72], or electronic nose [73]. Finally, Boukid et al. [74] evaluated the effect of thermal treatments and the addition of ingredients in the physical properties of tomato double concentrate, another tomato product similar to tomato sauce. All the above-mentioned works used chemometric methods to analyze their data. The untargeted analyses proposed in the present work are simpler than the targeted chemical analyses and generally require a shorter analysis time despite yielding similar results, although in this case study, only the discriminations between brands and manufacturers were explored. Moreover, as already stated, no chemical reagents were used in the current work, making the analyses cheaper and cleaner. The untargeted methods coupled with chemometrics [72,73] are able to extract useful information with a lower analysis effort, optimizing times and costs without losing effectiveness. In addition, as shown for GC-IMS analysis in this work, and in line with the work of Vitalis et al. [72], an untargeted method can be carried out to obtain a fingerprint of the samples; then, with the help of chemometrics, some particularly interesting variables can be highlighted and deeply studied, without the need of an in-depth quantification of all the possible analytes. The untargeted methods, indeed, are not intended to replace the “classical” targeted ones, but to assist them in optimizing the analyses by focusing only on the most important analytes.

## 4. Conclusions

Multivariate analysis is often applied to wine, olive oil, honey, milk, and other food matrices in order to identify and prevent food adulteration, counterfeit, and fraud on geographical origin. Adulteration techniques are becoming increasingly more advanced, and a continuous optimization of chemical and physical analysis is needed.

Conventional approaches envision the use of HPLC and GC data for chemometric analysis in order to evaluate sample quality and verify label information; however, these techniques, require time, sample preparation, and most of all, the use of organic solvents which should be discouraged where possible to reduce waste and promote sustainable chemistry. For the analysis of tomato sauce, GC coupled to mass spectrometry and IMS has been previously applied. This work applied a multivariate approach on the volatile organic and colloidal profile of Italian tomato sauces: VOCs were analysed by GC coupled both to a flame ionisation detector (GC-FID) and to an ion mobility spectrometry (GC-IMS), the latter only aimed at molecules deemed characteristic via GC-FID-derived PCA. The colloidal fraction was instead analysed by asymmetric flow field-flow fractionation (AF4), which was applied to this type of sample for the first time. Untargeted analysis was used to collect fingerprints of the samples and explore the capability of these combined techniques to show clustering of different tomato sauce brands. Overall, the results allowed a complete characterisation of the food matrix and provided a better understanding of its complexity. Different combinations of GC and AF4 data showed promising grouping perspectives: interestingly, colloidal and volatile fractions, though very different in composition and type, offered similar grouping. The AF4 data were analysed by either the whole profile or colloidal ratio, yielding comparable or better results than GC in terms of quality while providing complementary information. The potential of this combined approach was demonstrated and offers a great advantage in classifying tomato sauces. The ability to work in saline conditions (AF4), with easy pretreatment (or no pretreatment in the case of GC, with an analysis time of less than 2 min) and no chemical waste, is a huge environmental advantage with respect to techniques such as HPLC or GC-MS. This combined approach should therefore be considered when designing experiments involving large numbers of samples.

## Figures and Tables

**Table 1:** Molecular attribution for the characteristic peaks obtained from tomato sauce analysis through GC-IMS highlighted in Figure 6; numbers correspond to those reported in Figure 6. A description of the compounds’ origin (if derived from a degradation process) or its flavour is provided with a corresponding reference.

Peak	Attribution	Flavour or Origin	Reference
1	Heptanal	Fat, citrus, rancid	[26]
2	Heptan-2-one	Fruity, spicy	[65]
3	Isovaleric acid	--	[26,28,66]
4	Hexanal	Grass, tallow, fat	[28]
5	Pentanal	Green, grass	[66,67]
6	Methyl-butanal	Cocoa, almond, malt	[26]
7	1-hexene	--	--
8	α-pinene	--	[67]
9	2-hexanone	Possible product of tomato fertilization	[68]
10	3-methyl-butan-1-ol	Whiskey, malt, burnt	[26]
11	2-methyl-butanol	Malt, wine, onion	[26]
12	Toluene	Carotenoids degradation	[69]
13	Dimethyl-sulphide	Thermal decomposition of tomato sauce	[66,70]
14	Iso-pentanal	Malt	[26]

## Data Availability

Not applicable.

## Supplementary Materials

The following supporting information can be downloaded at https://www.mdpi.com/article/10.3390/molecules27175507/s1, Figure S1: Representative AF4-multidetection output of tomato sauce; Figure S2: The UV-MALS of three representative samples of tomato sauce from manufacturers D, N, and I.

## Sample Availability

Samples of the compounds are not available from the authors.

## References

[1] (2022). Special Eurobarometer 389: Europeans’ Attitudes towards Food Security, Food Quality and the Countryside.

[2] Spink J., Bedard B., Keogh J., Moyer D.C., Scimeca J., Vasan A. (2019). International Survey of Food Fraud and Related Terminology: Preliminary Results and Discussion. J. Food Sci..

[3] Everstine K., Spink J., Kennedy S. (2013). Economically motivated adulteration (EMA) of food: Common characteristics of EMA incidents. J. Food Prot..

[4] Tibola C.S., da Silva S.A., Dossa A.A., Patrício D.I. (2018). Economically Motivated Food Fraud and Adulteration in Brazil: Incidents and Alternatives to Minimize Occurrence. J. Food Sci..

[5] Moore J.C., Spink J., Lipp M. (2012). Development and Application of a Database of Food Ingredient Fraud and Economically Motivated Adulteration from 1980 to 2010. J. Food Sci..

[6] Jackson L.S. (2009). Chemical Food Safety Issues in the United States: Past, Present, and Future. J. Agric. Food Chem..

[7] Antonella F., Maria N., Mario S. lo M. ISMEA I Numeri Dellafiliera del Pomodoro da Industria, Rome, Italy, June 2017. DirezioneServizi per lo Svilupporurale. https://www.ismea.it.

[8] Ministero Delle Politiche Agricole E Forestali (2006). Passata di Pomodoro. Origine del Pomodoro Fresco.

[9] Ministero Delle Politiche Agricole E Forestali (2020). Proroga delle Disposizioni Obbligatorie di Indicazione dell’Origine, in Etichetta, del Grano duro per Paste di Semola di Grano Duro, del Riso e dei Derivati del Pomodoro.

[10] Palianskikh A.I., Sychik S.I., Leschev S.M., Pliashak Y.M., Fiodarava T.A., Belyshava L.L. (2022). Development and validation of the HPLC-DAD method for the quantification of 16 synthetic dyes in various foods and the use of liquid anion exchange extraction for qualitative expression determination. Food Chem..

[11] Arvanitoyannis I.S., Vaitsi O.B. (2007). A Review on Tomato Authenticity: Quality Control Methods in Conjunction with Multivariate Analysis (Chemometrics). Crit. Rev. Food Sci. Nutr..

[12] Médina B., Salagoïty M.H., Guyon F., Gaye J., Hubert P., Guillaume F. (2013). Using New Analytical Approaches to Verify the Origin of Wine.

[13] Slimani S., Bultel E., Cubizolle T., Herrier C., Rousselle T., Livache T. (2020). Opto-Electronic Nose Coupled to a Silicon Micro Pre-Concentrator Device for Selective Sensing of Flavored Waters. Chemosensors.

[14] Rasekh M., Karami H., Fuentes S., Kaveh M., Rusinek R., Gancarz M. (2022). Preliminary study non-destructive sorting techniques for pepper (*Capsicum annuum* L.) using odor parameter. LWT.

[15] Son H.S., Hwang G.S., Ahn H.J., Park W.M., Lee C.H., Hong Y.S. (2009). Characterization of wines from grape varieties through multivariate statistical analysis of 1H NMR spectroscopic data. Food Res. Int..

[16] Khorramifar A., Rasekh M., Karami H., Malaga-Toboła U., Gancarz M.A. (2021). Machine Learning Method for Classification and Identification of Potato Cultivars Based on the Reaction of MOS Type Sensor-Array. Sensors.

[17] Marengo E., Mazzucco E., Robotti E., Gosetti F., Manfredi M., Calabrese G. (2016). Characterization Study of Tomato Sauces Stored in Different Packaging Materials. Curr. Anal. Chem..

[18] Song H., Liu J. (2018). GC-O-MS technique and its applications in food flavor analysis. Food Res. Int..

[19] Fragni R., Trifirò A., Nucci A., Seno A., Allodi A., Di Rocco M. (2018). Italian tomato-based products authentication by multi-element approach: A mineral elements database to distinguish the domestic provenance. Food Control.

[20] Arrizabalaga-Larrañaga A., Epigmenio-Chamú S., Santos F.J., Moyano E. (2021). Determination of banned dyes in red spices by ultra-high-performance liquid chromatography-atmospheric pressure ionization-tandem mass spectrometry. Anal. Chim. Acta.

[21] Rusinek R., Gawrysiak-Witulska M., Siger A., Oniszczuk A., Ptaszyńska A.A., Knaga J., Malaga-Toboła U., Gancarz M. (2021). Effect of Supplementation of Flour with Fruit Fiber on the Volatile Compound Profile in Bread. Sensors.

[22] Zappi A., Melucci D., Scaramagli S., Zelano A., Marcazzan G.L. (2018). Botanical traceability of unifloral honeys by chemometrics based on head-space gas chromatography. Eur. Food Res. Technol..

[23] Eiceman G.A., Karpas Z., Hill H.H. (1990). Ion mobility spectrometry. Anal. Chem..

[24] Cumeras R., Figueras E., Davis C.E., Baumbach J.I., Gràcia I. (2015). Review on Ion Mobility Spectrometry. Part 1: Current instrumentation. Analyst.

[25] Buttery R.G., Ling L.C. (1993). Volatile Components of Tomato Fruit and Plant Parts. Bioact. Volatile Compd. Plants.

[26] Li J., Di T., Bai J. (2019). Distribution of Volatile Compounds in Different Fruit Structures in Four Tomato Cultivars. Molecules.

[27] Wang L., Qian C., Bai J., Luo W., Jin C., Yu Z. (2018). Difference in volatile composition between the pericarp tissue and inner tissue of tomato (*Solanum lycopersicum*) fruit. J. Food Process. Preserv..

[28] Servili M., Selvaggini R., Taticchi A., Begliomini A.L., Montedoro G. (2000). Relationships between the volatile compounds evaluated by solid phase microextraction and the thermal treatment of tomato juice: Optimization of the blanching parameters. Food Chem..

[29] Ali M.Y., Sina A.A.I., Khandker S.S., Neesa L., Tanvir E.M., Kabir A., Khalil M.I., Gan S.H. (2020). Nutritional Composition and Bioactive Compounds in Tomatoes and Their Impact on Human Health and Disease: A Review. Foods.

[30] Raffo A., Leonardi C., Fogliano V., Ambrosino P., Salucci M., Gennaro L., Bugianesi R., Giuffrida F., Quaglia G. (2002). Nutritional value of cherry tomatoes (*Lycopersicon esculentum* Cv. Naomi F1) harvested at different ripening stages. J. Agric. Food Chem..

[31] Opara U.L., Al-Ani M.R., Al-Rahbi N.M. (2012). Effect of Fruit Ripening Stage on Physico-Chemical Properties, Nutritional Composition and Antioxidant Components of Tomato (*Lycopersicum esculentum*) Cultivars. Food Bioprocess Technol..

[32] Luthria D.L., Mukhopadhyay S., Krizek D.T. (2006). Content of total phenolics and phenolic acids in tomato (*Lycopersicon esculentum* Mill.) fruits as influenced by cultivar and solar UV radiation. J. Food Compos. Anal..

[33] Kader A., Morris L.L., Stevens M.A., Albright Holton M. (1978). Composition and flavor quality of fresh market tomatoes as influenced by some postharvest handling procedures. J. Am. Soc. Hortic. Sci..

[34] Marassi V., Roda B., Casolari S., Ortelli S., Blosi M., Zattoni A., Costa A.L., Reschiglian P. (2018). Hollow-fiber flow field-flow fractionation and multi-angle light scattering as a new analytical solution for quality control in pharmaceutical nanotechnology. Microchem. J..

[35] Marassi V., Roda B., Zattoni A., Tanase M., Reschiglian P. (2014). Hollow fiber flow field-flow fractionation and size-exclusion chromatography with MALS detection: A complementary approach in biopharmaceutical industry. J. Chromatogr. A.

[36] Marassi V., Casolari S., Roda B., Zattoni A., Reschiglian P., Panzavolta S., Tofail S.A.M., Ortelli S., Delpivo C., Blosi M. (2015). Hollow-fiber flow field-flow fractionation and multi-angle light scattering investigation of the size, shape and metal-release of silver nanoparticles in aqueous medium for nano-risk assessment. J. Pharm. Biomed. Anal..

[37] Zattoni A., Roda B., Borghi F., Marassi V., Reschiglian P. (2014). Flow field-flow fractionation for the analysis of nanoparticles used in drug delivery. J. Pharm. Biomed. Anal..

[38] Marassi V., Di Cristo L., Smith S.G.J., Ortelli S., Blosi M., Costa A.L., Reschiglian P., Volkov Y., Prina-Mello A. (2018). Silver nanoparticles as a medical device in healthcare settings: A five-step approach for candidate screening of coating agents. R. Soc. Open Sci..

[39] Marassi V., Beretti F., Roda B., Alessandrini A., Facci P., Maraldi T., Zattoni A., Reschiglian P., Portolani M. (2019). A new approach for the separation, characterization and testing of potential prionoid protein aggregates through hollow-fiber flow field-flow fractionation and multi-angle light scattering. Anal. Chim. Acta.

[40] Wankar J., Bonvicini F., Benkovics G., Marassi V., Malanga M., Fenyvesi E., Gentilomi G.A., Reschiglian P., Roda B., Manet I. (2018). Widening the Therapeutic Perspectives of Clofazimine by Its Loading in Sulfobutylether β-Cyclodextrin Nanocarriers: Nanomolar IC 50 Values against MDR S. epidermidis. Mol. Pharm..

[41] Marassi V., Giordani S., Reschiglian P., Roda B., Zattoni A. (2022). Tracking Heme-Protein Interactions in Healthy and Pathological Human Serum in Native Conditions by Miniaturized FFF-Multidetection. Appl. Sci..

[42] Marassi V., Casolari S., Panzavolta S., Bonvicini F., Gentilomi G.A., Giordani S., Zattoni A., Reschiglian P., Roda B. (2022). Synthesis Monitoring, Characterization and Cleanup of Ag-Polydopamine Nanoparticles Used as Antibacterial Agents with Field-Flow Fractionation. Antibiotic.

[43] Roda B., Marassi V., Zattoni A., Borghi F., Anand R., Agostoni V., Gref R., Reschiglian P., Monti S. (2018). Flow field-flow fractionation and multi-angle light scattering as a powerful tool for the characterization and stability evaluation of drug-loaded metal–organic framework nanoparticles. Anal. Bioanal. Chem..

[44] Marassi V., Calabria D., Trozzi I., Zattoni A., Reschiglian P., Roda B. (2021). Comprehensive characterization of gold nanoparticles and their protein conjugates used as a label by hollow fiber flow field flow fractionation with photodiode array and fluorescence detectors and multiangle light scattering. J. Chromatogr. A.

[45] Marassi V., Maggio S., Battistelli M., Stocchi V., Zattoni A., Reschiglian P., Guescini M., Roda B. (2021). An ultracentrifugation—Hollow-fiber flow field-flow fractionation orthogonal approach for the purification and mapping of extracellular vesicle subtypes. J. Chromatogr. A.

[46] Marassi V., De Marchis F., Roda B., Bellucci M., Capecchi A., Reschiglian P., Pompa A., Zattoni A. (2021). Perspectives on protein biopolymers: Miniaturized flow field-flow fractionation-assisted characterization of a single-cysteine mutated phaseolin expressed in transplastomic tobacco plants. J. Chromatogr. A.

[47] Ventouri I.K., Loeber S., Somsen G.W., Schoenmakers P.J., Astefanei A. (2022). Field-flow fractionation for molecular-interaction studies of labile and complex systems: A critical review. Anal. Chim. Acta.

[48] Nilsson L. (2013). Separation and characterization of food macromolecules using field-flow fractionation: A review. Food Hydrocoll..

[49] Coelho C., Parot J., Gonsior M., Nikolantonaki M., Schmitt-Kopplin P., Parlanti E., Gougeon R.D. (2017). Asymmetrical flow field-flow fractionation of white wine chromophoric colloidal matter. Anal. Bioanal. Chem..

[50] Marassi V., Marangon M., Zattoni A., Vincenzi S., Versari A., Reschiglian P., Roda B., Curioni A. (2021). Characterization of red wine native colloids by asymmetrical flow field-flow fractionation with online multidetection. Food Hydrocoll..

[51] Osorio-Macías D.E., Bolinsson H., Linares-Pastén J.A., Ferrer-Gallego R., Choi J., Peñarrieta J.M., Bergenståhl B. (2022). Characterization on the impact of different clarifiers on the white wine colloids using Asymmetrical Flow Field-Flow Fractionation. Food Chem..

[52] Guyomarc’H F., Violleau F., Surel O., Famelart M.H. (2010). Characterization of heat-induced changes in skim milk using asymmetrical flow field-flow fractionation coupled with multiangle laser light scattering. J. Agric. Food Chem..

[53] Lie-Piang A., Leeman M., Castro A., Börjesson E., Nilsson L. (2021). Revisiting the dynamics of proteins during milk powder hydration using asymmetric flow field-flow fractionation (AF4). Curr. Res. Food Sci..

[54] Abbate R.A., Raak N., Boye S., Janke A., Rohm H., Jaros D., Lederer A. (2019). Asymmetric flow field flow fractionation for the investigation of caseins cross-linked by microbial transglutaminase. Food Hydrocoll..

[55] Krebs G., Gastl M., Becker T. (2021). Chemometric modeling of palate fullness in lager beers. Food Chem..

[56] Pascotto K., Leriche C., Caillé S., Violleau F., Boulet J.C., Geffroy O., Levasseur-Garcia C., Cheynier V. (2021). Study of the relationship between red wine colloidal fraction and astringency by asymmetrical flow field-flow fractionation coupled with multi-detection. Food Chem..

[57] Geiss O., Bianchi I., Senaldi C., Barrero J. (2019). Challenges in isolating silica particles from organic food matrices with microwave-assisted acidic digestion. Anal. Bioanal. Chem..

[58] Melucci D., Bendini A., Tesini F., Barbieri S., Zappi A., Vichi S., Conte L., Gallina T.T. (2016). Rapid direct analysis to discriminate geographic origin of extra virgin olive oils by flash gas chromatography electronic nose and chemometrics. Food Chem..

[59] Morozzi P., Zappi A., Gottardi F., Locatelli M., Melucci D. (2019). A quick and efficient non-targeted screening test for saffron authentication: Application of chemometrics to gas-chromatographic data. Molecules.

[60] Forleo T., Zappi A., Gottardi F., Melucci D. (2020). Rapid discrimination of Italian Prosecco wines by head-space gas-chromatography basing on the volatile profile as a chemometric fingerprint. Eur. Food Res. Technol..

[61] Dong W., Tan L., Zhao J., Hu R., Lu M. (2015). Characterization of Fatty Acid, Amino Acid and Volatile Compound Compositions and Bioactive Components of Seven Coffee (*Coffea robusta*) Cultivars Grown in Hainan Province, China. Molecules.

[62] Bro R., Smilde A.K. (2014). Principal component analysis. Anal. Methods.

[63] Nijhuis A., De Jong S., Vandeginste B.G.M. (1997). Multivariate statistical process control in chromatography. Chemom. Intell. Lab. Syst..

[64] Granato D., Santos J.S., Escher G.B., Ferreira B.L., Maggio R.M. (2018). Use of principal component analysis (PCA) and hierarchical cluster analysis (HCA) for multivariate association between bioactive compounds and functional properties in foods: A critical perspective. Trends Food Sci. Technol..

[65] Koltun S.J., MacIntosh A.J., Goodrich-Schneider R.M., Klee H.J., Hutton S.F., Sarnoski P.J. (2021). Sensory and chemical characteristics of tomato juice from fresh market cultivars with comparison to commercial tomato juice. Flavour Fragr. J..

[66] Vallverdú-Queralt A., Bendini A., Tesini F., Valli E., Lamuela-Raventos R.M., Toschi T.G. (2013). Chemical and sensory analysis of commercial tomato juices present on the Italian and Spanish markets. J. Agric. Food Chem..

[67] Socaci S.A., Socaciu C., Mureşan C., Fǎrcaş A., Tofanǎ M., Vicaş S., Pintea A. (2014). Chemometric discrimination of different tomato cultivars based on their volatile fingerprint in relation to lycopene and total phenolics content. Phytochem. Anal..

[68] Wright D.H., Harris N.D. (1985). Effect of Nitrogen and Potassium Fertilization on Tomato Flavor. J. Agric. Food Chem..

[69] Rios J.J., Fernández-García E., Mínguez-Mosquera M.I., Pérez-Gálvez A. (2008). Description of volatile compounds generated by the degradation of carotenoids in paprika, tomato and marigold oleoresins. Food Chem..

[70] Buttery R.G., Teranishi R., Ling L.C., Turnbaugh J.G. (1990). Quantitative and Sensory Studies on Tomato Paste Volatiles. J. Agric. Food Chem..

[71] Lo Feudo G., Naccarato A., Sindona G., Tagarelli A. (2010). Investigating the Origin of Tomatoes and Triple Concentrated Tomato Pastes through Multielement Determination by Inductively Coupled Plasma Mass Spectrometry and Statistical Analysis. J. Agric. Food Chem..

[72] Vitalis F., Zaukuu J.-L.Z., Bodor Z., Aouadi B., Hitka G., Kaszab T., Zsom-Muha V., Gillay Z., Kovacs Z. (2020). Detection and Quantification of Tomato Paste Adulteration Using Conventional and Rapid Analytical Methods. Sensors.

[73] Mohammad-Razdari A., Ghasemi-Varnamkhasti M., Yoosefian S.H., Izadi Z., Siadat M. (2019). Potential application of electronic nose coupled with chemometric tools for authentication assessment in tomato paste. J. Food Process. Eng..

[74] Boukid F., Curti E., Diantom A., Carini E., Vittadini E. (2021). A multilevel investigation supported by multivariate analysis for tomato product formulation. Eur. Food Res. Technol..

