# Supplementary material for: A Green Analytical Method Combined with Chemometrics for Traceability of Tomato Sauce Based on Colloidal and Volatile Fingerprinting
Source: Molecules. 2022 Aug 27;27(17):5507. doi: 10.3390/molecules27175507 (PMC9457838; doi:10.3390/molecules27175507)
Supplement: Supplementary file 1 [file molecules-27-05507-s001.zip › molecules-1842694-supplementary.pdf]

molecules

MDPI

Article

A Green Analytical Method Combined with Chemometrics for Traceability of Tomato Sauce Based on Colloidal and Volatile Fingerprinting

Alessandro Zappi[1], Valentina Marassi[1,2,*], Nicholas Kassouf[1], Stefano Giordani[1], Gaia Pasqualucci[1], Davide Garbini[3], Barbara Roda[1,2], Andrea Zattoni[1,2], Pierluigi Reschiglian[1,2], Dora Melucci[1,4]

* Correspondence: valentina.marassi@unibo.it

Supplementary Material

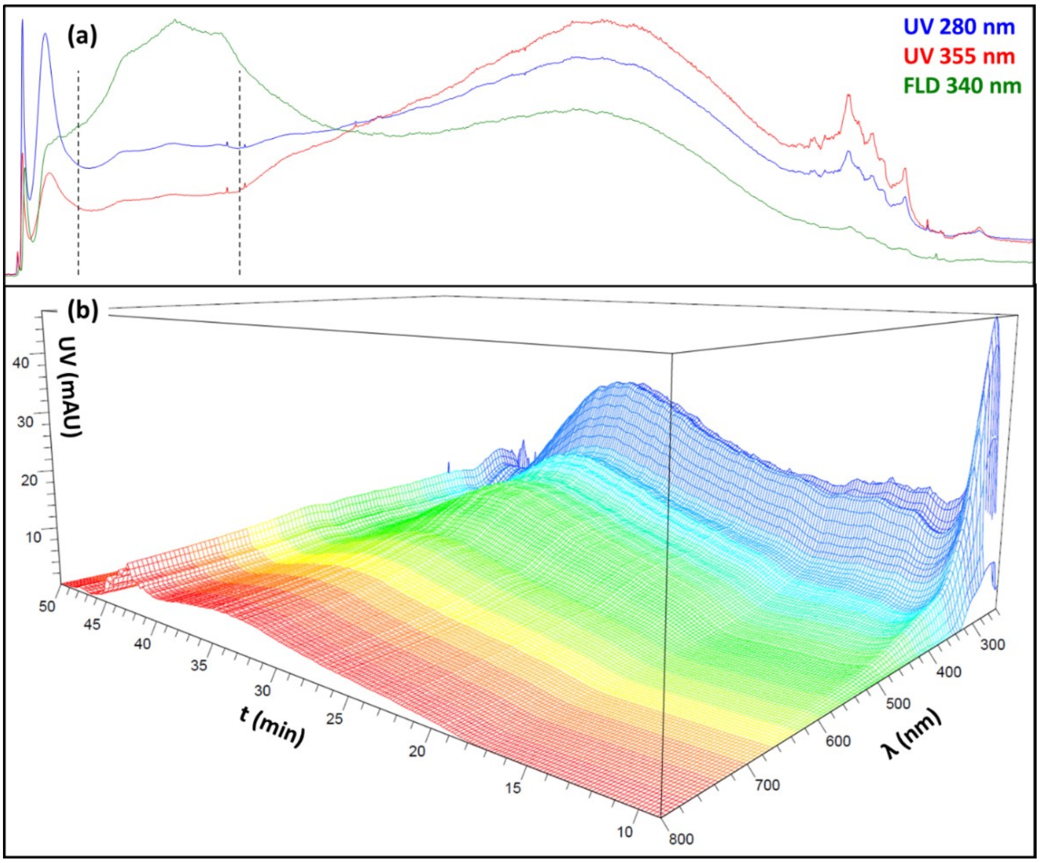

Figure S1. Representative AF4-multidetection output of tomato sauce: **a)** overlay of UV absorption (at 280 nm, blue; and at 355 nm, red) and Fluorescence emission (excitation at 280 nm, emission at 340 nm, green)—dashed lines: splitting in the three bands for PCA analysis; and **b)** 3D UV absorption spectrum.

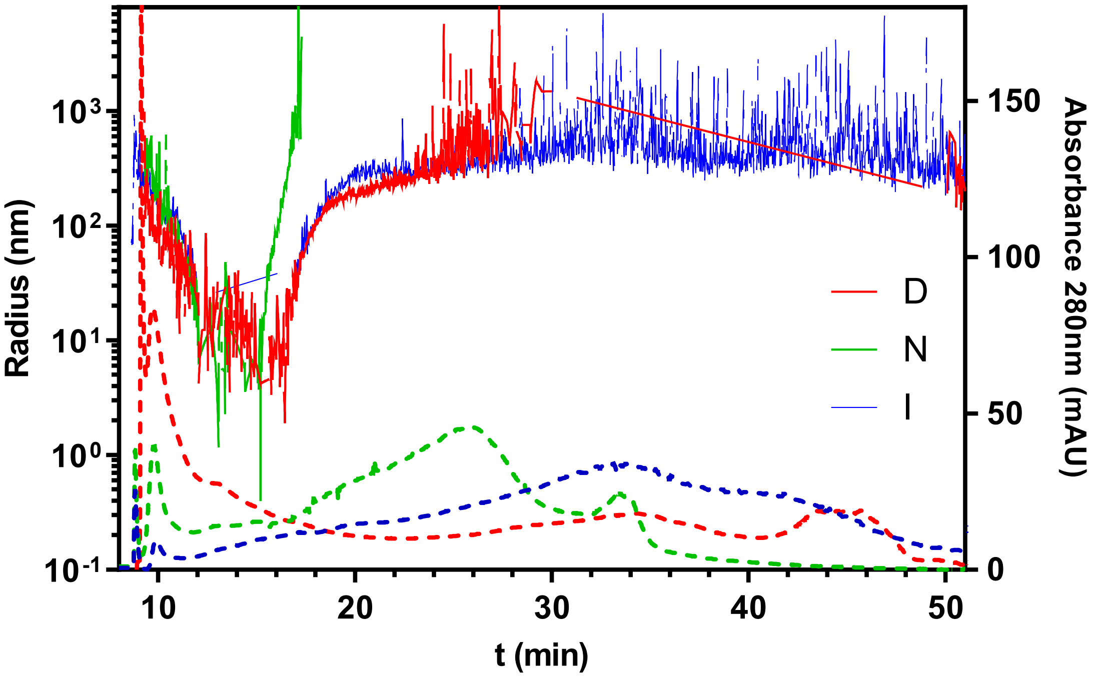

Figure S2. The UV-MALS of three representative samples of tomato sauce from manufacturers D, N, and I. Dashed lines: UV fractogram at 280 nm. Solid lines: Gyration radius calculation in the range 10-1000 nm.
